# Dataset of hydropower characteristics for creating ramping and generation constraints in aggregated energy system models across European bidding zones

**DOI:** 10.1016/j.dib.2026.113132

**Published:** 2026-08-04

**Authors:** Julia Kiehle, Tomi J. Lindroos, Jean-Nicolas Louis, Eva Pongrácz

**Affiliations:** a University of Oulu, Faculty of Technology, Water, Energy and Environmental Engineering, Pentti Kaiteran katu 1, P.O. Box 4300, FI-90014, Oulu, Finland; b VTT Technical Research Centre of Finland Ltd, Energy, Tekniikantie 21, Espoo, 02044, Finland

**Keywords:** Europe, Energy system modelling, Flexibility, Aggregated hydropower, Pondage volume

## Abstract

The dataset was created to support the implementation of ramping and generation constraints for hydropower in large-scale aggregated energy system models. It is based on collected and subsequently processed data from ENTSO-E’s transparency platform, but can be freely applied to models utilising other data sources. Historical generation data builds the basis for the dataset and has been processed into weekly data series for hourly maximum and minimum generation, as well as maximum hourly ramping rates. The proposed constraints are available for different types of hydropower, pumped storage, reservoir and run-of-river, aggregated in the European bidding zones. In addition, one subset of the dataset focuses on the pondage of run-of-river hydropower plants, aiming to provide estimates of storage volumes based on historical cumulative outputs. The dataset was used in [1] to create the operational framework for aggregated hydropower, enabling analysis of lost flexibility at the European scale.

Specifications Table

**Table:** 

Subject	Engineering & Materials science
Specific subject area	Hydropower representation in large-scale energy system models
Type of data	Table, Graph, Excel -files Analyzed, Processed
Data collection	The data was collected from publicly available data repositories and subsequently processed. Collection parameters include the type of hydropower plant, bidding zones and period of time.
Data source location	Primary data source: ENTSO-E Transparency platform: https://transparency.entsoe.eu/ Actual generation per production type (Hydro Pumped Storage, Hydro Run-of-river and pondage, Hydro Water Reservoir), 2020–2024, 44 bidding zones
Data accessibility	Repository name: Zenodo Data identification number: doi:10.5281/zenodo.19,091,702 Direct URL to data: https://zenodo.org/records/19091702
Related research article	J. Kiehle, T.J. Lindroos, J.-N. Louis, E. Pongrácz. (2026) Lost flexibility: Hydropower capabilities in large-scale, pan-European energy system models. Applied Energy 2026; 423:128,324. https://doi.org/10.1016/J.APENERGY.2026.128324.

## 1 Value of the Data

• The value of the data lies in supporting the implementation of constraints to hydropower representation based on historically observed operating ranges, preventing over- or underestimation of operational flexibility available. The dataset provides time series data and annual values for hourly-scale maximum and minimum electricity production and ramping rates over the course of a year, derived from processed historical electricity market data.
• Energy system model researchers and model users can utilise the dataset to create ramping and generation constraints, based on historical performance, for aggregated hydropower in (large-scale, pan-European) energy system models. The dataset supports defining constant and time-step-dependent constraints for reservoir plants, pumped hydro storage units and run-of-river plants with pondage. The main goal is to improve the accuracy of aggregated models, which are usually unable to depict the multi-plant and multi-reservoir dynamics depicted in disaggregated models.
• Implementing hydropower constraints in aggregated models prevents overestimation of available generation capacity or underestimation of minimum output compared to historical operational data. Limiting available ramping rates counteracts the typical bias of highly aggregated models of extreme ramps between consecutive hours, forcing more realistic ramping behaviour.
• The representation of run-of-river plants fully dependent on influx without the ability to react to market signals is common but potentially inaccurate. Missing information about pondage volumes limits the application of more accurate modelling approaches. A subset of this dataset can be used to estimate the pondage storage volume of run-of-river hydropower plants, reducing potential underestimation of their flexible operation. The data is based on historically observed operations and is indicative.
• The derived data is especially relevant to large-scale, highly aggregated energy system models used for studying energy systems with high penetration of variable renewable energy sources. It offers a low-threshold approach to improve the accuracy of hydropower representation, which is often limited by data availability and computational load, without employing complex disaggregated model setups. Whereas hydrologically accurate, disaggregated models remain superior in hydropower accuracy, this approach balances modelling bias and complexity.

## 2 Background

The creation of the dataset aimed at increasing the accuracy of representing hydropower plants in aggregated energy system models. Hydropower is a valuable flexibility provider in power systems [1], but highly aggregated setups tend to overestimate the flexible operational potential of hydropower [2,3]. Detailed representation of dynamics among individual power plants is often infeasible to implement for pan-continental, large-scale energy system models due to a lack of granular non-proprietary data. Furthermore, run-of-river hydropower plants are often modelled as inflexible electricity sources due to a lack of information on pondage volumes, even though studies have shown that this approach leads to an underestimation of their potential [4]. Based on lessons learned from modelling cascading hydropower systems at a national scale, the objective was to address the lack of unified datasets for hydropower operational constraints in aggregated, large-scale energy system models.

The data article supplements the article by Kiehle et al. [5], describing the creation of the datasets in more detail and discussing their utilisation. The dataset is model-agnostic and can be used by any energy system model that seeks to account for hydropower dynamics. The goal is to make the created datasets available to a wider audience and disassociate them from the case study presented in the published article.

## 3 Data Description

The dataset includes two individual subsets:

(1) Data in .xlsx format for creating ramping, maximum and minimum generation constraints for bidding zone-wise aggregated hydropower plants, including pumped hydro storage (PHS), run-of-river hydropower with pondage (ROR) and reservoir hydropower (RES) plants (HydropowerConstraints_NominalValues.xlsx & HydropowerConstraints_RelativeValues.xlsx).
(2) Data in .xlsx format listing estimated pondage volumes for run-of-river hydropower aggregated per bidding zone (ROR_Storage.xlsx)

Both subsets focus on European bidding zones as listed on ENTSO-E’s transparency platform.

Subset 1 consists of two files in .xlsx format: HydropowerConstraints_NominalValues.xlsx and HydropowerConstraints_RelativeValues.xlsx. The first sheet in both files (“Overview”) lists installed capacities per hydropower type and bidding zone, subject to availability. The same sheet includes an overview table of nominal constraint values, showing annual maximum and minimum hourly generation limits (MW) and maximum hourly ramping rates (MW/h) in absolute and relative format. Absolute values are based solely on processed historical generation data, while relative values are calculated relative to the installed capacity. The latter has been added to the dataset due to mismatches and contradictions in hydropower information across data sources: Absolute values might not fit models using sources other than ENTSO-E’s transparency platform. Subsequently, the HydropowerConstraints_NominalValues.xlsx file presents maximum and minimum observed historical hourly generation values, aggregated weekly, along with the observed maximum hourly ramping rate. The file then offers a “LookUp” sheet, allowing the selection of a specific bidding zone and hydropower type to visualise the weekly-aggregated operational data and the suggested model constraints.

Furthermore, the “LookUp” sheet provides visualisation of suggested constraints compared to the operational data. The presented nominal values can be used directly if the chosen model’s input data matches the dataset's data source. In other cases, the dataset offers the option to apply relative constraints to model-specific installed capacities. These relative constraints are presented in the same format as the nominal values in the HydropowerConstraints_RelativeValues.xlsx file. This includes a “LookUp” sheet for an easier overview of the available constraints per bidding zone and hydropower type. The dataset can be utilised to create both constant and time-series-based constraints, reflecting the historically observed operating ranges of European hydropower. Table 1, Table 2 list the relative values usable for the creation of generation and ramping rate constraints for RES and ROR plants.

**Table 1:** Overview of bidding zone-aggregated operational hydropower constraints for run-of-river plants with pondage [% of installed capacity].

**Bidding zone**	**Maximum hourly generation**	**Minimum hourly generation**	**Maximum hourly ramping rate**
	% of installed capacity	% of installed capacity	% of installed capacity
AT	92	23	14
BE	29	0	26
BG	78	0	24
CH	77	7	25
CZ	84	0	47
DE_LU	61	21	18
DK_1	73	0	38
EE	43	0	37
ES	76	10	20
FI	81	10	23
FR	76	0	50
HR	86	0	47
HU	61	3	32
LT	82	11	57
LV	92	0	39
NO_2	84	8	34
NO_3	78	0	30
NO_4	78	1	19
NO_5	77	1	28
PL	83	9	22
RO	83	4	22
RS	97	1	61
SI	91	0	75
XK	72	0	34
**Bidding zones subject to limitations**			
BA^1^	(100)	0	77
IE^2^	(100)	0	18
IT_CALA^2^	(100)	0	73
IT_CNOR^2^	(100)	1	33
IT_CSUD^2^	(100)	5	33
IT_NORD^2^	(100)	5	24
IT_SUD^2^	(100)	0	74
MD^2^	(100)	0	98
NO_1^1^	(100)	10	37
SK^2^	(100)	0	67
**Bidding zones excluded from this subset**			
NL^3^	-	-	-
PT^3^	-	-	-

1 Mismatch between reported capacity and observed hourly generation on ENTSO-E’s transparency platform (maximum hourly generation replaces capacity, see Limitations).

2 Missing installed capacity on ENTSO-E’s transparency platform (maximum hourly generation replaces capacity).

3 Missing historical generation data on ENTSO-E’s transparency platform, exclusion from this subset marked with “-“.

**Table 2:** Overview of bidding zone-aggregated operational hydropower constraints for reservoir hydropower plant [% of installed capacity].

**Bidding zone**	**Maximum hourly generation**	**Minimum hourly generation**	**Maximum hourly ramping rate**
	% of installed capacity	% of installed capacity	% of installed capacity
AT	69	0	30
BG	86	0	49
CH	72	1	35
DE_LU	71	0	36
ES	61	1	22
FR	58	0	17
GR	84	0	42
HR	90	0	36
HU	88	0	31
NO_2	93	2	22
NO_3	99	8	28
NO_4	94	7	42
NO_5	89	4	27
PL	60	0	45
RO	72	0	31
RS	70	0	57
UA	66	0	43
**Bidding zones subject to limitations**			
BA^1^	(100)	0	85
CZ^1^	(100)	0	67
IT_CALA^2^	(100)	0	72
IT_CNOR^2^	(100)	0	69
IT_CSUD^2^	(100)	0	84
IT_NORD^2^	(100)	0	47
IT_SUD^2^	(100)	0	73
NO_1^1^	(100)	3	34
SE_1^2^	(100)	5	31
SE_2^2^	(100)	10	33
SE_3^2^	(100)	5	39
SE_4^2^	(100)	2	54
SK^2^	(100)	0	71
**Bidding zones excluded from this subset**			
PT^3^	-	-	-
XK^3^	-	-	-

1 Mismatch between reported capacity and observed hourly generation on ENTSO-E’s transparency platform (maximum hourly generation replaces capacity, see Limitations).

2 Missing installed capacity on ENTSO-E’s transparency platform (maximum hourly generation replaces capacity).

3 Missing historical generation data on ENTSO-E’s transparency platform, exclusion from this subset marked with “-“.

**Fig. 1:**
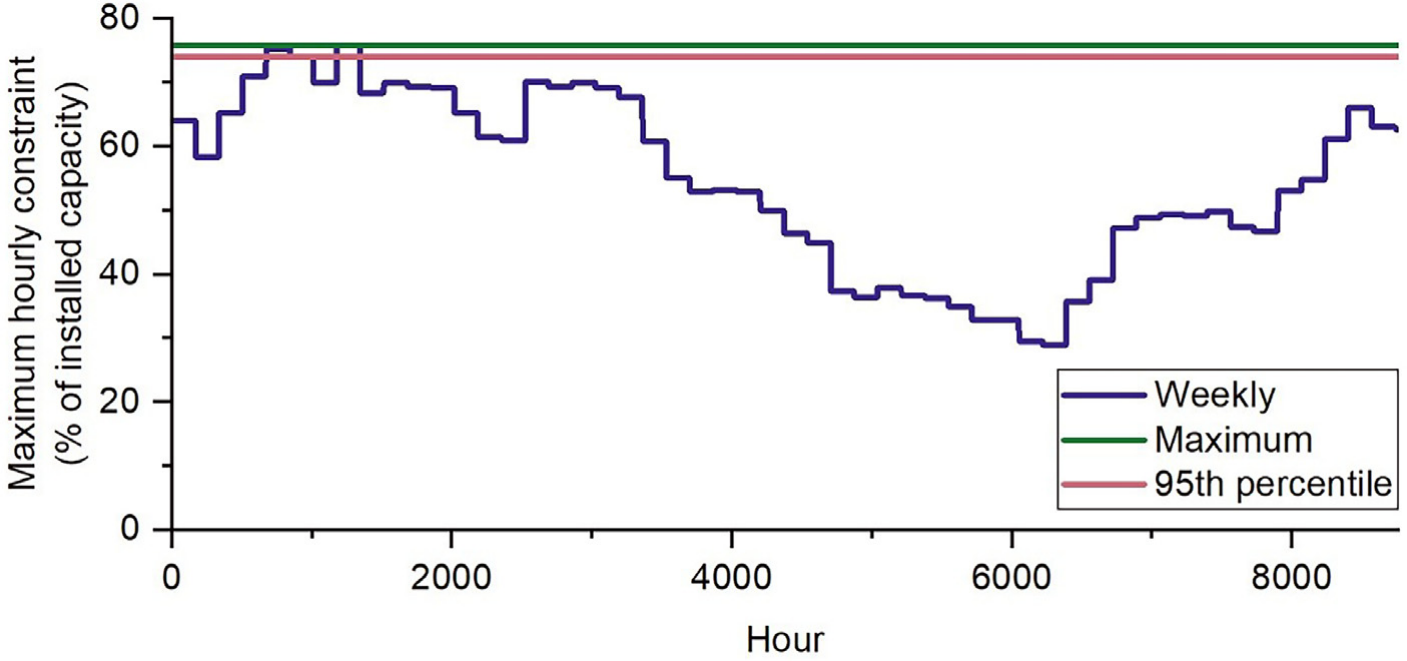
Maximum hourly constraint (restricted production) for run-of-river hydropower in Spanish bidding zone – week-dependent time series and two constant constraints (maximum value and 95th percentile).

**Fig. 2:**
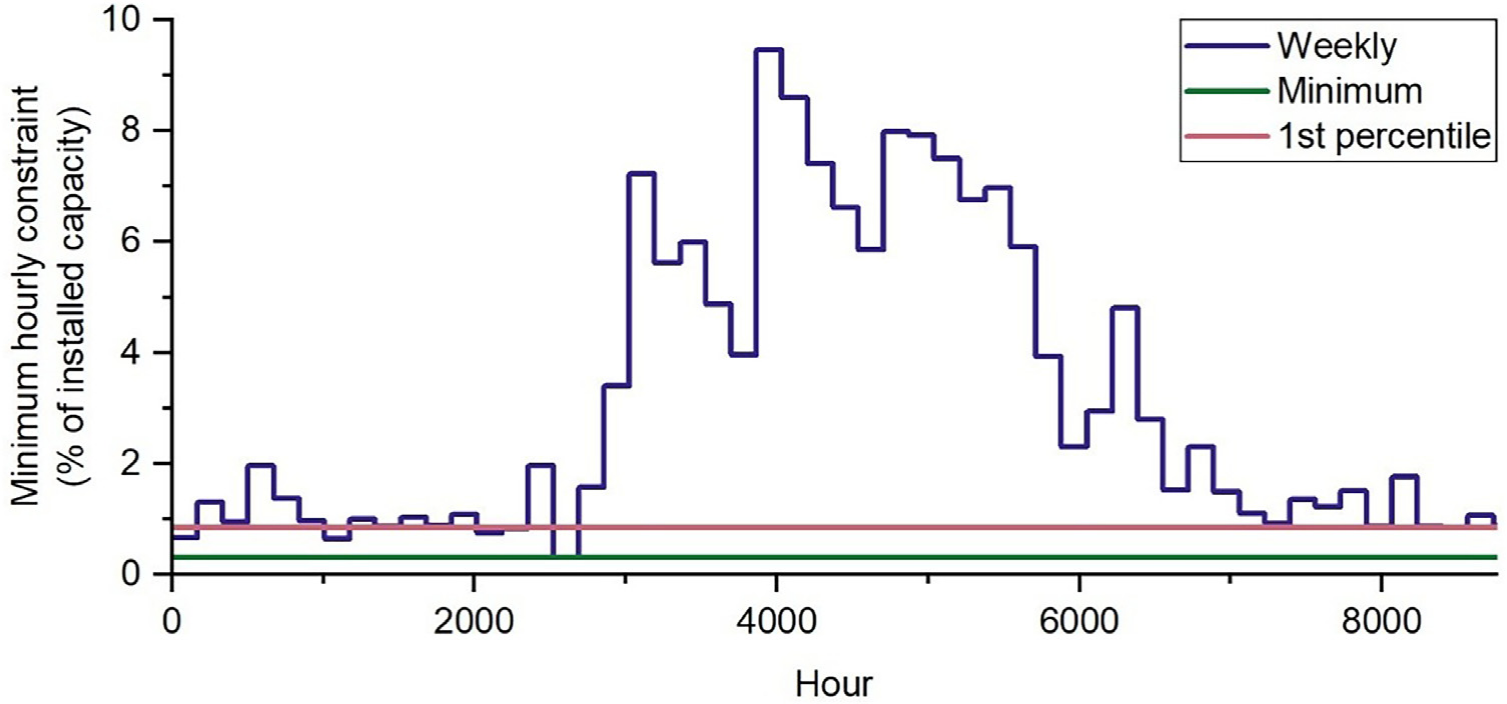
Minimum hourly constraint (forced production) for reservoir hydropower in Austrian bidding zone – week-dependent time series and two constant constraints (minimum value and 1st percentile).

**Fig. 3:**
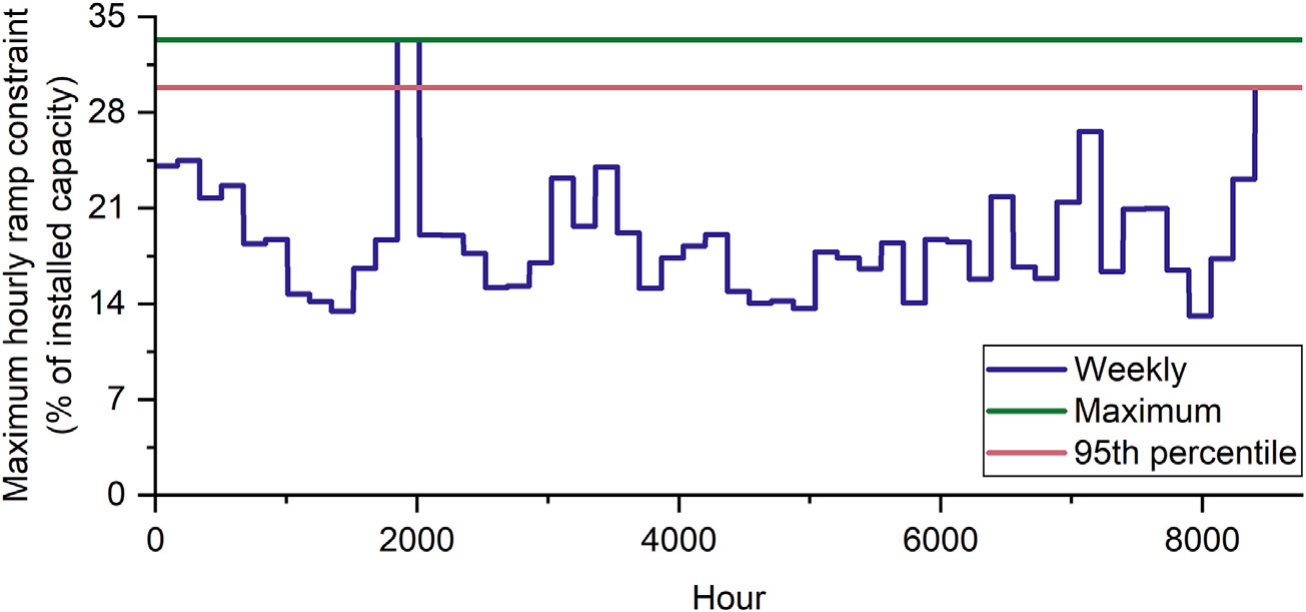
Maximum hourly ramp constraint (restricted ramping) for reservoir hydropower in Swedish bidding zone SE02 – week-dependent time series and two constant constraints (maximum value and 95th percentile).

Absolute values can be used to implement constant, year-round operational constraints for hydropower plants. The dataset also provides data to create weekly time series as a basis for time-dependent constraints. The constraint data includes the maximum/minimum values and 95th/1st percentile values, offering different levels of restrictiveness. Fig. 1, Fig. 2, Fig. 3 show example cases for weekly-changing and constant constraint values for maximum and minimum hourly generation, as well as hourly ramping rates. These types of graphs are available for each plant type and bidding zone in the dataset’s “LookUp” sheets.

Use-case example of relative constraint values: The maximum hourly constraint for Spanish run-of-river hydropower plants is 76% of installed capacity. Based on data from ENTSO-E’s transparency platform, the installed capacity is 2629 MW in 2026 [6]. This results in an absolute generation constraint of 1998 MW. In contrast, utilising ENTSO-E’s hydropower modelling data published for the Midterm Adequacy Forecast 2019 (MAF2019) [7], an installed capacity of 3300 MW would lead to a generation constraint of 2508 MW. This way, the dataset ensures its usability in different model setups. Furthermore, an aggregated model solely based on installed capacities would overestimate the available hourly generation capacity of Spanish ROR plants by 631–792 MW, depending on the model’s data source. If the model setup further allows ramping between extreme outputs (near-zero to full capacity), Spanish ROR plants would be depicted as significantly more flexible than their historical behaviour indicates: the dataset lists 20% of capacity as the maximum observed ramping rate. This indicates an overestimated ramping potential of 2103 and 2640 MW/h for transparency platform data and MAF2019, respectively. In addition to using the dataset to compute constant constraints, the weekly time-series data can be used to create time-step-dependent restrictions. As shown in Fig. 1, this would considerably decrease the allowed maximum generation further, especially between time steps 4000 and 8000. Compared to 76% of installed capacity as constant, variable constraints could incorporate the drop to below 40% around time step 6000, reflecting the changed availability of hydropower generation during a year.

Subset 2 provides estimated storage volumes for bidding zone-wise aggregated run-of-river hydropower. ROR plants are commonly defined as units with storage volumes unable to accommodate the mean daily influx [8]. The pondage estimation allows the modelling of ROR plants with limited flexibility, which would not be possible without storage volume. The volume is given in hours of full-capacity output. This unit of storage volume has been chosen as a substitute for a relative value, allowing the application of the dataset’s values to various data sources. It reflects how many hours a ROR plant can run at full capacity before the pondage volume available for hydropower operation is depleted. The dataset provides different pondage sizes depending on the calculation method, allowing for individual adjustments across various model setups. Table 3 shows storage volumes per bidding zone, calculated using the 24-h and 48-h cumulative output relative to the maximum hourly generation.

**Table 3:** Estimated pondage volume (in hours of full-capacity output) for run-of-river hydropower plants aggregated per bidding zone, calculated with two different methods.

Bidding zone	Storage volume	
	24-h cumulative output	48-h cumulative output
	Maximum generation	Maximum generation
	[hours of full-capacity output]	[hours of full-capacity output]
AT	1.75	4.57
BA	1.57	3.93
BE	3.36	8.84
BG	1.7	3.9
CH	2.24	5.15
CZ	1.89	4.3
DE_LU	1.71	4.26
DK1	1.71	4.56
EE	1.32	3.6
ES	1.19	3.13
FI	3.34	7.14
FR	1.78	4.31
HR	2.65	6.66
HU	2.3	6.03
IT_CALA	2.02	3.67
IT_CNOR	1.43	4.17
IT_CSUD	1.74	4.71
IT_NORD	1.48	2.95
IT_SUD	2.23	4.96
LT	4	7.4
LV	2.51	4.56
MD	3.15	7.61
NO_1	0.75	2.88
NO_2	1.73	4.66
NO_3	2.13	6.18
NO_4	1.89	4.92
NO_5	3.2	8.16
PL	1.2	3.14
RO	1.73	4.02
RS	1.52	3.34
SI	3.06	7.6
SK	2.26	6.39
XK	2.63	8.12
**Bidding zones excluded from this subset**		
PT^1^	-	-

1 Missing historical generation data on ENTSO-E’s transparency platform, exclusion from this subset marked with “-“.

The ROR pondage dataset also includes a use-case example that shows how the values can be applied to different data sources by multiplying the hour-based storage volume and the installed capacity. The estimated storage volume for the Austrian ROR plants totals 4.57 h of full-capacity output (based on 48-h cumulative output, maximum generation). Based on ENTSO-E’s MAF2019 data [7], the installed capacity of Austrian ROR plants is 5817 MW. This results in a storage volume of 26.6 GWh. Utilising a different calculation approach changes the estimated pondage volume: Based on the 24-h cumulative output, the volume is 10.2 GWh. The differing calculation approaches are intended to provide the user with options and to indicate the uncertainty around this estimation. While not fully conclusive, the dataset offers indicative results for ROR pondage volume within European bidding zones.

For a comprehensive overview, see the dataset published in Zenodo [9].

## 4 Experimental Design, Materials and Methods

The primary data source for the raw data, further processed to create the dataset, is ENTSO-E’s transparency platform [6]. Historical hourly generation data for hydropower, aggregated by bidding zone, was obtained from there. The data comprises the years 2020–2024, features 44 European bidding zones, and has been allocated to three hydropower plant types. In total, the combination of bidding zones and plant types resulted in 94 individual generation series. The featured types follow the definition of the transparency platform: “Pumped hydro storage plant” (PHS), “run-of-river hydropower with pondage” (ROR) and “reservoir hydropower” (RES). Both subsets of the dataset are based on the same raw data.

Subset 1, providing data on the operational constraints for hydropower, was created by calculating weekly-aggregated data series based on the obtained 5-year hourly generation data (2020–2024). This first step yielded a time series spanning 261 weeks. Those data series were created for the hourly minimum and maximum generation output, as well as the hourly ramping rate. The latter has been calculated as the absolute difference between the outputs of two consecutive hours. To reduce the impact of outliers and data errors, the 99th and 1st percentiles were applied to the weekly data for maximum and minimum values, respectively. The next step was to create a weekly representation of an average year by aggregating 261 weeks of data into one year’s 53 weeks. This was done for each of the 94 individual generation series and for all three constraint options. After those steps, the processed data series consisted of absolute values based on the transparency platform data. The calculation approach for the maximum hourly generation is defined by Eq. (1). Eq. (2) shows the procedure utilised to calculate the minimum hourly generation, and Eq. (3) provides details on the ramp rate calculation.

(1) $$E_{w,max}=\;\max_{y}\left(q_{0.99}\left({\left\{E_{y,w,h}\right\}}_{h=1}^{n}\right)\right)$$

where $E_{w,max}$ is the maximum value of the weekly 99th -percentile hourly energy generation that occurs over all years y∈{2020,2021,2022,2023,2024} for each week w∈{1,…,52}, where $E_{y,w}$ is the weekly 99th -percentile value of hourly energy generation, n is the total number of considered hours n∈{168}, and $E_{y,w,h}$ is the hourly generation for year y, week w, hour h.

(2) $$E_{w,min}=\;\min_{y}\left(q_{0.01}\left({\left\{E_{y,w,h}\right\}}_{h=1}^{n}\right)\right)$$

where $E_{w,min}$ is the minimum value of the weekly 1st -percentile hourly energy generation that occurs over all years y for each week w.

(3) $$R_{w,max}^{\Delta }=\;\max_{y}\left(q_{0.99}\left({\left\{\;\left|\Delta E_{y,w,h}\right|\;\right\}}_{h=2}^{n}\right)\right)$$

where $\Delta E_{y,w,h}$ is the hour-to-hour ramping rate for year y, week w, hour h, and $R_{w,max}^{\Delta }$ is the maximum value of the weekly 99th -percentile hourly ramping rates over the years y for each week w.

To ensure the data’s usability also in relation to other data sources, relative data series were created in a subsequent step. The aggregated, weekly absolute values for the minimum and maximum hourly generation (MW) and the maximum hourly ramping rate (MW/h) were set relative to the installed capacity of the individual hydropower type and bidding zone. The results are data series in “% of installed capacity” that can be freely applied to specific installed capacities reported by different data sources. Based on the created data series, both absolute and relative, overall annual values were calculated, representing constant constraints applicable to the aggregated hydropower categories in each bidding zone. The values also include 95th-percentile values for maximum constraints and 1st-percentile values for minimum constraints as alternative options.

Due to missing or mismatched values in the raw data, some corrective measures were implemented. For bidding zones without reported installed capacities, the calculated hourly maximum generation output was used to create the data series with relative values. In case of mismatched values, e.g., a higher maximum hourly generation than installed capacity, the calculated hourly generation limit was applied to produce the relative data series. In addition, due to the shifting numbering of weeks in a year, week 53 had fewer data points in the raw data, leading to outliers at the end of the year. For the ramping constraints, week 53 has been excluded for all bidding zone-hydropower type-combinations and replaced with the value for week 52. Conversely, data for maximum and minimum generation constraints were handled on a case-by-case basis: If week 53 caused a considerable spike, it was excluded and replaced with week 52 data to avoid skewing the constraints. For maximum generation, the cut-off criterion was an output difference (relative to installed capacity) between weeks 52 and 53 higher than 25%. Week 53 data for the minimum generation were replaced if the spike would have increased the largest minimum value or if the output difference was above 10%. The following zones have undergone this treatment:

- Maximum generation: EE, IT_CSUD (ROR, RES), LT (PHS, ROR), NO_5 (ROR), PL (PHS, ROR), SE_1, SK (ROR, RES), UA, XK (ROR)
- Minimum generation: BA (RES), BE (ROR), DK_1, EE, ES (RES), FI, FR (PHS, ROR), HR (RES), HU, LT (ROR), ME, NO_1 (RES), NO_2 (ROR, RES), NO_3 (RES), NO_5 (PHS, RES)

Subset 2, estimating pondage volumes of aggregated run-of-river hydropower, was created by calculating the moving cumulative deviation of electricity output based on the 5-year generation data (2020–2024) obtained from ENTSO-E’s transparency platform. Two different time scales were utilised for calculating the cumulative output: 24 h and 48 h. First, the rolling average for the specific time scale was calculated for each bidding zone. At each hour of the year, the average values over the last 24 or 48 hourly observations were obtained. With each step forward, older values were discarded. This moving average was then compared with the hourly output at each time step to compute the deviation between them. These hourly deviation values were subsequently summed according to the chosen time scale. This means that when using a 48-hour time scale, the rolling average accounts for two days, and the summed deviation comprises 48 consecutive hours to arrive at the final value. The cumulative volume in MWh was calculated using the 99.5th percentile of the generated data series. The exclusive percentile was chosen to exclude potential extreme outliers, accounting for the 44 h with the highest values. This percentile value was then set in relation to the observed maximum output, resulting in storage volumes in hours of full-capacity output. These storage volumes can be combined with installed capacity data from different sources to estimate the pondage volume of aggregated run-of-river hydropower in MWh. The calculation of the cumulative output is described in Eqs. (4)–6 for a 48-hour window.

(4) $$RAO_{h}^{\left(48\right)}=\;\frac{1}{48}\sum_{i=h-47}^{h}E_{i},\;h\geq 48$$

(5) $$p_{0.995}=q_{0.995}\left({\left\{\sum_{i=h-47}^{h}\left(E_{i}-RAO_{i}^{\left(48\right)}\right)\right\}}_{h=48}^{H}\right)$$

(6) $$V_{MO}=\frac{p_{0.995}}{E_{MO}}$$

where $RAO_{h}^{\left(48\right)}$ is the rolling average output with a 48-hour window [MWh], $E_{i}$ is the energy output in hour i [MWh], $p_{0.995}$ is the 99.5th percentile of the cumulative deviation distribution [MWh], $E_{MO}$ is the maximum observed output between 2020 and 2024 [MW] and $V_{MO}$ is the storage volume [h of full-capacity output].

Subsequently, the storage volume in hours of full-capacity output can be multiplied by the installed capacity of a hydropower unit [MW], resulting in a storage volume in MWh independent of whether the dataset’s primary data source and the chosen modelling data source match. As there are no comprehensive measured pondage-volume data for European ROR plants, a superior estimation method cannot be offered. Therefore, two different estimated values are provided, allowing users to select the most appropriate method for their application.

## Limitations

The dataset comprises constraints developed to limit hydropower operation in large-scale, highly aggregated energy system models based on historically observed operating ranges. It is important to emphasise that this historical behaviour does not reflect technical limitations. The scheduling of hydropower plants is determined by the conditions across various electricity markets, the rules in place (such as environmental flow constraints), and the hydrological conditions in a given climate year. Future energy systems might require different operational strategies for hydropower plants. However, aggregated hydropower representation tends to be biased towards extreme and rapid transitions between full capacity and near-zero output instead of continuous but steady ramping (see, for example, the following studies: [2,3,5]). While such behaviour might be technically possible, it is rarely observed in such quantities as modelled by aggregated setups. The proposed constraints could be described as probabilistic: excluding possible but rare extreme scheduling behaviour and ensuring that less-likely operational decisions are not the modelled standard operation. Disaggregated representation of hydropower remains the more accurate modelling approach, but these constraints offer a low-threshold approach to improve accuracy. The target audience is energy system modellers not focusing on hydropower, but modelling systems that rely on hydropower flexibility and are therefore subject to impacts from over- or underestimating hydropower operational ranges.

The estimated pondage volumes for ROR plants should be interpreted as indicative values, rather than validated storage capacities. The main motivation was to fill the gap left by missing publicly available data of European ROR plants. Therefore, the values represent a first attempt to provide bidding-zone-level estimates suitable for aggregated energy system models. Due to the lack of available statistical data, the individual estimates cannot be systematically verified against pondage volumes. Further, the pondage capacity of specific ROR plants may differ significantly from the calculated values. However, the dataset focuses on providing estimations for aggregated groups of hydropower plants within a bidding zone, not individual plants. The two estimation approaches are intended to reflect uncertainty, rather than provide alternative representations of a known quantity. This allows modellers to conduct sensitivity analyses and adapt different assumptions to the characteristics of their own modelling framework. Where local hydropower information is available, users are encouraged to calibrate the values accordingly. While individual verification was not possible, a model setup utilising the data has been tested and verified in [5]. The resulting behaviour was found to be consistent with the intended representation of aggregated ROR hydropower operation.

The primary data source included some missing and mismatched data, which limited the ability to provide a fully comprehensive dataset. Installed capacity was not reported for 13 of 44 bidding zones, and the listed capacity did not match the reported maximum hourly generation in 4 zones. In those cases, the maximum hourly generation value was applied as supplementary installed capacity. This allowed the creation of capacity-related ramping constraints, but it may misrepresent reality. Historical generation data was missing for 2 bidding zones, resulting in their effective exclusion from the dataset. For the following bidding zones, historical generation data was not available for the full targeted five-year period (2020–2024) from ENTSO-E’s transparency platform: BA, IE (ROR), IT_CALA, MD, NO_2 (PHS), NO_3 (PHS), NO_5 (PHS), SE_1, SE_2, SE_3, SE_4, UA and XK.

## Ethics Statement

The authors have read and followed the ethical requirements for publication in Data in Brief and confirm that the current work does not involve human subjects, animal experiments, or any data collected from social media platforms.

## CRediT Author Statement

**Julia Kiehle:** Conceptualisation, Methodology, Software, Validation, Formal analysis, Data curation, Visualisation, Writing – Original Draft. **Tomi J. Lindroos:** Conceptualisation, Methodology, Software, Validation, Formal analysis, Writing – Review & Editing. **Jean-Nicolas Louis:** Conceptualisation, Methodology, Writing – Review & Editing, Supervision. **Eva Pongrácz:** Writing – Review & Editing, Supervision, Funding acquisition.

## Data Availability

ZenodoHydropower dataset for creating operational constraints in aggregated energy system models covering European bidding zones (Original data)
